# Annotations

**Published:** 1910-05-07

**Authors:** 


### ANNOTATIONS.

### Vaccination in Scotland.

In his report on "Vaccination" in Scotland in 1909, which has just been issued, the Registrar-General of Births, Deaths, and Marriages says that in the three years before the Vaccination (Scotland) Act 1907 came into operation, successfully vaccinated children in Scotland amounted to 93.46 per cent. of the children surviving at the time of compulsory vaccination, and unvaccinated children to only 6.45 per cent.; last year the vaccinated children amounted to 76.13 per cent., and the unvaccinated to 23.87 per cent. On the assumption that the Act alone is accountable for the change, it may, according to these figures, be credited with adding fully 20,000 unvaccinated children to the population in one year.

### Surgical Diagnosis for the Laity.

A modest brochure which has reached us from Mr. Fisher Unwin ("Appendicitis," by Dr. Baumgärtner; 1s. net) marks a new departure in popular therapeutic literature. The attempt made by this German surgeon is to educate the public into an intelligent appreciation not merely of what appendicitis is, but also of how and why its potentialities are so dangerous and by what means it is best treated. With German thoroughness, a short sketch of the anatomy of the viscera and peritoneum is given, together with some elementary pathology and remarks on diagnosis. It cannot be denied that the layman capable of comprehending these sections must be an individual very difficult to find; some of the language is unduly technical, and this, so far as can be judged, is apparently not the fault of the translator, whose work has been done very well. There follow details—harrowing details, in fact, to non-professional readers—of many severe cases of appendicitis, including large abscesses, gangrene, perforation, and the other complications met with in severe forms of the disease. The moral pointed throughout is the enormous importance of surgical intervention when once the symptoms have declared themselves; the whole aim and object of the publication seems to be to induce the laity to submit readily and with confidence to such treatment, and to educate them to an appreciation of some of the leading features of the morbid processes and the effects they produce. It is freely illustrated with photographs of grossly diseased appendices, which again can convey but little to any but medical men. The idea is one to be applauded, though we entertain a slight scepticism as to the suitability of the form in which Dr. Baumgärtner has dressed it for the public, at any rate in England.

### The Diet of the Chinese.

In a memoir to the Belgian Academy of Medicine, Dr. Spruyt, physician to the Imperial Chinese Railway Company, gives an account of his observations with regard to the diet of the inhabitants of Central China. Having under his care groups of men whose diet he could supervise for long periods of time, he took the opportunity of estimating the food values of the rations usually consumed. Since no exact analysis of the food was made, and only the quantities consumed noted, his figures can only be compared with those of authors who deal with diet in the raw and take no account of waste and loss due to the excreta. Such as they are, his figures show that the diet of the inhabitants of Central China hardly differs from that of the Central European peoples. The proportion of albumins, carbohydrates, and fats consumed by these Celestials are quite analogous to that which forms the diet of the Central European. So far from being a vegetarian, as popularly supposed, the Chinaman living in parts remote from the coast, and hence removed from the influence of Western ideas, consumes pork, game, mutton, dog's-flesh, goat's-flesh, and eggs in abundance. These last in an advanced state of decomposition constitute a very *recherché* dish. Neither milk, cheese, nor beef find a place in his dietary, and rice and sorghum form an important and even preponderating element in the daily menu, but this last is none the less varied. In Canton, dogs are fattened for the butcher, and throughout the empire fish is a staple dish. It is no more true to say that a Chinaman lives entirely on rice than to maintain that an Irishman lives exclusively on potatoes.

### A Benevolent Hercules.

The following anecdote concerning Dr. Gallard, at one time chief physician to a hospital and to a railway company, and noted in the profession for his herculean strength, is reported in the *Centre Médical*. In the year 1874, on a winter's evening, a travelling acrobat, shivering in his spangled tights, was endeavouring to interest a large crowd near the Pont d'Austerlitz, in Paris, in his exhibition of weight-lifting. His obvious discouragement reflected the poor monetary response made by the spectators to his efforts, and his spleen was further roused by his happening to overhear the conversation of two individuals in the crowd, one of whom, on being asked by the other if he could do as much as the professional, replied that he could do even better. "Come along, then, you lazy beggar!" shouted the acrobat. Whereupon there emerged from among the spectators a well-groomed individual, with a decoration in his button-hole, gloves on his hands, an overcoat on his arm, a cigar in his mouth, and a smile on his lips. Taking a five-franc piece from his pocket, he placed it in the hollow of an overturned 50-lb. weight, and lifting the latter in one hand, carried it at arm's length twice round the crowd, which increased at each moment. Having done this, he put down the weight which had served as a collecting-box in front of the professional Hercules, and immediately went off with his friend without disclosing his identity. The acrobat, in a dazed condition, counted over the collection that had been made in this amazing fashion, and found that he was the richer by 32 francs.

